# Construction and Validation of a Major Depression Risk Predictive Model for Patients with Coronary Heart Disease: Insights from NHANES 2005–2018

**DOI:** 10.31083/RCM25998

**Published:** 2025-01-13

**Authors:** Li-xiang Zhang, Shan-bing Hou, Fang-fang Zhao, Ting-ting Wang, Ying Jiang, Xiao-juan Zhou, Jiao-yu Cao

**Affiliations:** ^1^Department of Cardiology, The First Affiliated Hospital of USTC, Division of Life Science and Medicine, University of Science and Technology of China, 230001 Hefei, Anhui, China; ^2^Department of Emergency, The First Affiliated Hospital of USTC, Division of Life Science and Medicine, University of Science and Technology of China, 230001 Hefei, Anhui, China; ^3^Department of Rehabilitation Medicine, The First Affiliated Hospital of USTC, Division of Life Science and Medicine, University of Science and Technology of China, 230001 Hefei, Anhui, China

**Keywords:** coronary heart disease, major depression, NHANES, risk factors, predictive model

## Abstract

**Background:**

This study aimed to develop and validate a predictive model for major depression risk in adult patients with coronary heart disease (CHD), offering evidence for targeted prevention and intervention.

**Methods:**

Using data from the National Health and Nutrition Examination Survey (NHANES) from 2005 to 2018, 1098 adults with CHD were included. A weighted logistic regression model was applied to construct and validate a nomogram-based prediction tool for major depression in this population.

**Results:**

The weighted prevalence of major depression among these patients was 13.95%. Multivariate weighted logistic regression revealed that waist circumference, smoking status, arthritis, sleep disorders, and restricted work capacity were independent risk factors for major depression (odds ratio (OR) >1, *p* < 0.05). The areas under the receiver operating characteristic (ROC) curve in the nomogram model for both the development and validation cohorts were 0.816 (95% confidence interval (CI): 0.776–0.857) and 0.765 (95% CI: 0.699–0.832), respectively, indicating the model possessed strong discriminative ability. Brier scores in the development and validation cohorts were 0.107 and 0.127, respectively, both well below the 0.25 threshold, demonstrating good calibration. Decision curve analysis (DCA) showed that when the threshold probability for major depression ranged from 0.04 to 0.54 in the development group and from 0.08 to 0.52 in the validation group, the nomogram provided the highest clinical net benefit compared to “Treat All” and “Treat None” strategies, confirming its strong clinical utility.

**Conclusions:**

With a weighted prevalence of 13.95%, this nomogram model shows excellent predictive performance and clinical relevance for predicting major depression risk in patients with CHD. Thus, the model can be applied to aid healthcare professionals in identifying high-risk individuals and implementing targeted preventive strategies, potentially lowering the incidence of major depression in this patient population.

## 1. Introduction

With the advancement of modern society, factors such as the accelerating pace of life and the progression of population aging contribute to the steady rise in the annual incidence of cardiovascular diseases. Among these, coronary heart disease (CHD) stands out due to its high morbidity and mortality rates, posing a significant threat to public health [1]. As a chronic and debilitating condition, CHD is often accompanied by various comorbidities, prolonging its course and acting as a profound source of psychological stress for patients, frequently resulting in emotional disturbances, such as depression [2, 3, 4]. Negative emotions, particularly depression, have a considerable impact on the onset, progression, and quality of life of patients with CHD. There is strong evidence that depression is an independent risk factor for the development and prognosis of CHD [5, 6]. Despite this, current mainstream treatments for CHD, such as interventional, surgical, and pharmacological therapies, often neglect the psychological aspects of care, contributing to the high prevalence of depression [1]. Study has reported that 15% to 20% of patients with CHD suffer from depression, a rate 2 to 4 times higher than that in the general population [7]. Depression not only exacerbates health risks but also hampers the social reintegration of patients and increases the societal burden [7]. The interaction of CHD and depression worsen the occurrence and prognosis of CHD, which seriously threatens the quality of life of patients [8, 9]. The interplay between CHD and depression is thus a critical focus of ongoing clinical research. However, the precise mechanisms underlying CHD comorbidity with depression remain unclear, it may involve physiological mechanisms and psychosocial factors [10], and there is a notable lack of targeted prevention and intervention strategies to address depression risk in patients with CHD.

A nomogram is a graphical tool that predicts individual disease risk or prognosis by integrating multiple risk factors, enabling clinicians to more accurately assess the likelihood of clinical events and implement timely interventions [11]. Prior studies have demonstrated the utility of nomograms in predicting depression outcomes following deep brain stimulation in patients with Parkinson’s disease [12], as well as in assessing depression risk in hypertensive adults in the USA and in predicting post-stroke depression [13, 14]. However, there remains a substantial gap in the literature concerning the application of nomograms to predict depression risk, specifically in patients with CHD, both domestically and internationally. Therefore, this study aimed to address this gap; moreover, to our knowledge, the present study is the first to analyze data from the National Health and Nutrition Examination Survey (NHANES) to identify key risk factors for major depression in patients with CHD and to develop a personalized depression risk predictive model. The resulting model can be used with the aim of providing an effective screening tool for assessing the risk of major depression in this patient population.

## 2. Materials and Methods

### 2.1 Study Population

The data used in this study were obtained from the NHANES [15]—a comprehensive national cross-sectional survey conducted by the US Centers for Disease Control and Prevention (CDC) to assess the health and nutritional status of the American population. The NHANES protocol was approved by the Ethics Review Committee of the National Center for Health Statistics (NCHS), and all participants provided written informed consent before their inclusion [16].

**Fig. 1.**
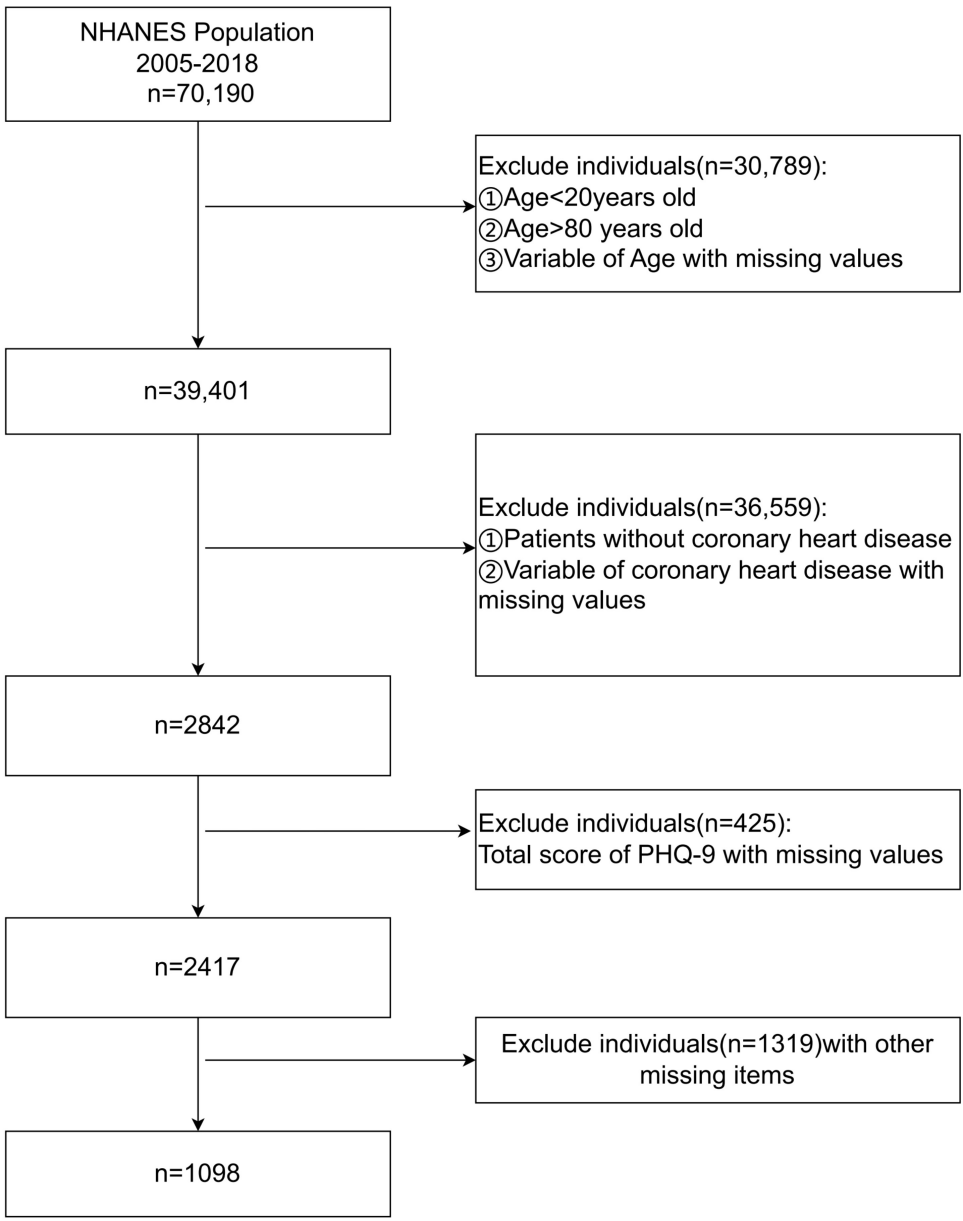
**Flowchart of the research participant screening process**. NHANES, the National Health and Nutrition Examination Survey; PHQ, Patient Health Questionnaire-9.

A total of 70,190 individuals were initially identified from the NHANES database covering the period from 2005 to 2018. The inclusion criteria for this analysis were (1) adults aged 20–80; (2) individuals diagnosed with CHD; (3) participants with a complete Patient Health Questionnaire-9 (PHQ-9) score; (4) those with complete data for all relevant study indicators. Exclusion criteria were (1) individuals younger than 20 or older than 80 or with missing age information; (2) those without a CHD diagnosis; (3) participants with incomplete or anomalous PHQ-9 scores; (4) those with missing data for other relevant indicators. After applying these criteria, 1098 individuals with CHD met the study requirements and were included. A detailed participant selection process flowchart is presented in Fig. 1.

### 2.2 Research-Related Indicators

The PHQ-9 comprises nine items and was used to screen for depression in the NHANES database and assess the frequency of depressive symptoms over the prior two weeks [17]. Each item offered four response options: “almost every day”, “more than half the days”, “several days”, and “never”, scored as 3, 2, 1, and 0 points, respectively. The total score was calculated by summing the individual scores, with a possible scoring range of 0 to 27. Participants scoring 10 or higher on the PHQ-9 were categorized as having major depression, while those with scores below 10 were not [18, 19]. CHD was defined as self-reported CHD, angina pectoris, or a history of myocardial infarction in the NHANES database [20].

Additionally, various demographic and health-related details were extracted from the NHANES database. Basic demographics included age, gender, education, family poverty–income ratio (PIR), marital status, health insurance coverage in the previous year, race, smoking status, alcohol consumption, recent anemia treatment (within three months), overweight status, sleep disorders, and physical activity levels. Physical measurements and laboratory data included height, weight, waist circumference, and hemoglobin levels. Comorbidities encompass hypertension, asthma, arthritis, congestive heart failure, stroke, emphysema, chronic bronchitis, liver disease, thyroid disorders, and diabetes. Gender was categorized as male or female. Education levels were grouped into high school or below, high school graduate/equivalent, some college/AA degree (Associate of Arts degree, a two-year undergraduate academic degree), and college graduate or higher.

The family PIR, representing the household income ratio to the poverty threshold, was based on guidelines from the US Department of Health and Human Services and categorized into less than 1, between 1 and 3, and greater than 3 [21]. Marital status was classified as married/living with a partner, widowed/divorced/separated, or never married. Racial categories included Mexican American, non-Hispanic White, other Hispanics, non-Hispanic Black, and other/multi-racial individuals. Smoking status was divided into daily, some days, or never, while alcohol consumption was categorized into yes or no. Sleep disorders were defined by self-reported difficulties [22]. The need for assistive walking devices was defined as having a walking impairment [22]. Work restrictions were defined as the inability to work due to chronic health conditions [22]. Body mass index (BMI) was calculated using anthropometric data collected by trained examiners at the Mobile Examination Center (MEC). Weight was measured in kilograms using a digital scale, and height in centimeters was measured using a stadiometer. BMI was then derived using the formula: BMI = weight (kg)/(height (m))^2^ [23]. Hypertension was defined as self-reported hypertension, current or previous use of antihypertensive medication, or an average systolic blood pressure of 130 mmHg or higher and/or an average diastolic blood pressure of 80 mmHg or higher [24]. Diabetes history was identified through self-reported diabetes, current diabetes treatment, or a glycosylated hemoglobin level of 6.5% or higher [25]. A random sampling method was applied to divide the dataset into development and validation groups. Each of the 1098 patient records was initially assigned a unique identifier. A random number of seeds of 123 ensured the randomization process was replicable. A random uniform variable was then generated for each patient, and the dataset was sorted according to this variable. From this, a simple random sample of 70% of the records was selected to form the development group. Specifically, the first 769 patients, as ordered by the random variable, were placed in the development group (coded as 1), while the remaining 329 patients constituted the validation group (coded as 2). The dataset was subsequently re-sorted using the original patient identifier to maintain its initial order. This procedure resulted in a development group comprising 70% of the dataset and a validation group representing the remaining 30%.

### 2.3 Statistical Analysis

Statistical analyses were performed using Stata (version 17.0, Stata Corp, TX, USA). Before the analysis, the data were adjusted using three NHANES weighting indicators: “SDMVPSU”, “SDMVSTRA”, and “WTMEC2YR”, to account for sampling variability and enhance the accuracy of results. Weighted measurement data conforming to a normal distribution were reported as the mean and standard deviation, and independent samples *t*-tests were used to compare the two groups. Categorical variables were presented as frequencies and percentages, with Pearson’s chi-square test used for group comparisons. Weighted univariate and multivariate logistic regression analyses were conducted to identify independent risk factors for major depression among patients with CHD. A nomogram model was developed to predict the likelihood of major depression in these patients. The discriminative power of the model was evaluated using the area under the receiver operating characteristic (ROC) curve, while its calibration was assessed using a calibration curve and Brier score. Clinical utility was evaluated via decision curve analysis (DCA). All statistical tests were two-tailed, and a *p*-value of less than 0.05 determined significance.

## 3. Results

### 3.1 Comparison of Patient Data between the Weighted Development Group and the Validation Group

The weighted mean age of the 1098 patients was 64.89 years, with a standard error of 0.440 years. Regarding gender distribution, 66.24% of the sample were men, and 33.76% were women. The weighted prevalence of major depression among patients with CHD was 13.95%. Additional descriptive statistics of the weighted data are provided in Table 1. No significant differences were observed between the weighted data of patients in the development and validation groups (*p* > 0.05), suggesting that the two groups were comparable and well-balanced, as outlined in Table 1.

**Table 1.** **Comparison of weighted data between the development group and the validation group**.

Variables		Total	Development group	Validation group	*p*-value^1^
N (Weighted)		1098 (5,621,690)	769 (3,991,457)	329 (1,630,233)	
Age (years), mean (SE)		64.89 (0.44)	64.66 (0.51)	65.45 (0.68)	0.323
BMI (kg/m^2^), mean (SE)		30.54 (0.30)	30.70 (0.33)	30.15 (0.44)	0.238
Waist circumference (cm), mean (SE)		108.17 (0.67)	108.30 (0.75)	107.87 (1.09)	0.726
Hemoglobin (g/dL), mean (SE)		14.30 (0.06)	14.24 (0.07)	14.45 (0.10)	0.066
Sleep time (hours), mean (SE)		7.13 (0.07)	7.17 (0.08)	7.03 (0.13)	0.345
Gender, % (SE)					0.110
	Male	66.24 (0.04)	64.49 (2.66)	70.53 (2.82)	
	Female	33.76 (0.03)	35.51 (2.66)	29.47 (2.82)	
Education level, % (SE)					0.090
	Below high school	23.84 (0.02)	23.32 (2.04)	25.12 (2.75)	
	High school graduation/equivalent	29.93 (0.03)	28.78 (2.47)	32.76 (3.31)	
	Some college or AA degree	31.12 (0.03)	33.93 (2.27)	24.26 (3.11)	
	College graduate or above	15.10 (0.02)	13.98 (1.94)	17.86 (3.06)	
Poverty–income ratio (PIR), % (SE)					0.449
	<1	15.92 (0.02)	14.99 (1.41)	18.22 (2.84)	
	1–3	44.16 (0.03)	44.03 (2.57)	44.47 (3.26)	
	>3	39.92 (0.03)	40.99 (2.59)	37.30 (3.80)	
Time without health insurance in the past year, % (SE)					0.611
	No	95.93 (0.05)	96.19 (0.93)	95.31 (1.60)	
	Yes	4.07 (0.01)	3.82 (0.93)	4.69 (1.60)	
Race, % (SE)					0.192
	Mexican American	2.62 (0.00)	2.44 (0.54)	3.05 (0.59)	
	Other Hispanics	3.20 (0.01)	2.64 (0.53)	4.55 (1.29)	
	Non-Hispanic Whites	80.67 (0.05)	81.37 (1.82)	78.94 (2.63)	
	Non-Hispanic Blacks	8.14 (0.01)	7.54 (0.92)	9.61 (1.64)	
	Other races (including multi-races)	5.38 (0.01)	6.01 (1.32)	3.85 (1.36)	
Smoke, % (SE)					0.076
	Daily	28.31 (0.02)	29.21 (2.10)	26.12 (3.15)	
	Some days	3.94 (0.01)	4.79 (1.09)	1.86 (0.65)	
	Never	67.75 (0.04)	66.00 (2.22)	72.02 (3.20)	
Marital status, % (SE)					0.334
	Never married	5.77 (0.01)	6.44 (1.31)	4.14 (1.52)	
	Married/living with a partner	62.08 (0.04)	62.67 (2.29)	60.65 (2.96)	
	Widowed/divorced/separated	32.15 (0.02)	30.90 (2.05)	35.21 (2.82)	
Complicated with hypertension, % (SE)					0.284
	No	19.59 (0.02)	18.46 (1.69)	22.36 (3.60)	
	Yes	80.41 (0.05)	81.54 (1.69)	77.64 (3.60)	
Asthma, % (SE)					0.164
	No	77.01 (0.04)	75.58 (2.25)	80.52 (2.72)	
	Yes	22.99 (0.02)	24.42 (2.25)	19.48 (2.72)	
Anemia treatment in the past 3 months, % (SE)					0.103
	No	94.01 (0.05)	93.22 (1.41)	95.95 (1.06)	
	Yes	5.99 (0.01)	6.78 (1.41)	4.05 (1.06)	
Overweight, % (SE)					0.373
	No	44.92 (0.03)	43.68 (2.38)	47.97 (4.21)	
	Yes	55.08 (0.04)	56.32 (2.38)	52.03 (4.21)	
Arthritis, % (SE)					0.216
	No	40.96 (0.03)	39.31 (2.36)	44.98 (3.76)	
	Yes	59.04 (0.04)	60.69 (2.36)	55.02 (3.76)	
Congestive heart failure, % (SE)					0.636
	No	73.64 (0.04)	74.16 (1.91)	72.36 (3.18)	
	Yes	26.37 (0.02)	25.84 (1.91)	27.64 (3.18)	
Stroke, % (SE)					0.519
	No	84.69 (0.05)	85.35 (1.42)	83.07 (3.47)	
	Yes	15.31 (0.02)	14.65 (1.42)	16.93 (3.47)	
Emphysema, % (SE)					0.800
	No	85.11 (0.05)	85.39 (1.97)	84.40 (3.55)	
	Yes	14.89 (0.02)	14.61 (1.97)	15.60 (3.55)	
Chronic bronchitis, % (SE)					0.113
	No	82.81 (0.05)	81.30 (1.97)	86.50 (2.15)	
	Yes	17.19 (0.02)	18.70 (1.97)	13.50 (2.15)	
Liver disease, % (SE)					0.384
	No	91.70 (0.05)	91.03 (1.66)	93.33 (1.77)	
	Yes	8.30 (0.01)	8.97 (1.66)	6.68 (1.77)	
Thyroid problems, % (SE)					0.575
	No	84.79 (0.05)	85.30 (1.59)	83.55 (2.79)	
	Yes	15.21 (0.02)	14.70 (1.59)	16.45 (2.79)	
Sleep disorder, % (SE)					0.651
	No	54.48 (0.03)	53.89 (2.31)	55.92 (4.05)	
	Yes	45.52 (0.03)	46.11 (2.31)	44.08 (4.05)	
Complicated with diabetes, % (SE)					0.224
	No	64.76 (0.04)	66.11 (2.00)	61.45 (3.57)	
	Yes	35.24 (0.03)	33.89 (2.00)	38.55 (3.57)	
Work restriction, % (SE)					0.577
	No	64.06 (0.04)	63.48 (2.27)	65.46 (3.12)	
	Yes	35.94 (0.03)	36.52 (2.27)	34.54 (3.12)	
Walking disorder, % (SE)					0.523
	No	75.47 (0.04)	74.84 (1.95)	77.02 (2.98)	
	Yes	24.53 (0.02)	25.17 (1.95)	22.98 (2.98)	
Drink wine/alcohol, % (SE)					0.914
	No	21.05 (0.02)	20.95 (2.01)	21.30 (2.74)	
	Yes	78.95 (0.044)	79.05 (2.01)	78.70 (2.74)	
Major depression, % (SE)					0.934
	No	86.05 (0.05)	85.98 (1.69)	86.21 (2.34)	
	Yes	13.95 (0.02)	14.02 (1.69)	13.79 (2.34)	

^1^Independent sample *t*-test, Pearson chi-square test. SE, standard error of the mean; AA degree, Associate of Arts degree, a two-year undergraduate academic degree; BMI, body mass index.

### 3.2 Results of Weighted Univariate Logistic Regression Analysis of Major Depression in the Development Group

The results of the weighted univariate logistic regression analysis in the development group identified sixteen indicators as significant predictors for major depression in patients with CHD (*p* < 0.05). These factors included age, BMI, waist circumference, sleep duration, gender, educational level, poverty–income ratio, smoking status, asthma, arthritis, congestive heart failure, stroke, chronic bronchitis, sleep disorders, work limitations, and walking impairments. A detailed summary of these findings is presented in Table 2.

**Table 2.** **Results of weighted univariate logistic regression analysis of major depression in the development group**.

Characters		Estimate	Std. error	*p*-value	OR	95% CI
Age		–0.042	0.011	<0.001	0.959	0.959 (0.939, 0.979)
BMI		0.065	0.019	<0.001	1.067	1.067 (1.028, 1.107)
Waist circumference		0.022	0.008	0.010	1.023	1.023 (1.006, 1.040)
Hemoglobin		–0.032	0.073	0.663	0.969	0.969 (0.837, 1.120)
Sleep time		–0.182	0.088	0.041	0.833	0.833 (0.700, 0.992)
Gender						
	Male	ref	ref	ref	ref	ref
	Female	0.754	0.258	0.004	2.125	2.125 (1.272, 3.549)
Education level						
	Below high school	ref	ref	ref	ref	ref
	High school graduation/equivalent	–0.409	0.354	0.252	0.665	0.665 (0.329, 1.344)
	Some college or AA degree	–0.105	0.324	0.746	0.9	0.900 (0.473, 1.713)
	College graduate or above	–1.736	0.442	<0.001	0.176	0.176 (0.073, 0.425)
Poverty–income ratio						
	<1	ref	ref	ref	ref	ref
	1–3	–0.784	0.307	0.013	0.456	0.456 (0.248, 0.841)
	>3	–1.833	0.446	<0.0001	0.16	0.160 (0.066, 0.389)
Time without health insurance in the past year						
	No	ref	ref	ref	ref	ref
	Yes	0.888	0.473	0.064	2.43	2.430 (0.948, 6.229)
Race						
	Mexican American	ref	ref	ref	ref	ref
	Other Hispanics	0.217	0.567	0.703	1.242	1.242 (0.402, 3.837)
	Non-Hispanic Whites	–0.166	0.456	0.716	0.847	0.847 (0.342, 2.096)
	Non-Hispanic Blacks	0.324	0.501	0.520	1.382	1.382 (0.510, 3.746)
	Other races (including multi-races)	0.159	0.518	0.760	1.172	1.172 (0.418, 3.285)
Smoke						
	Daily	ref	ref	ref	ref	ref
	Some days	–0.948	0.667	0.159	0.388	0.388 (0.103, 1.461)
	Never	–1.299	0.315	<0.0001	0.273	0.273 (0.146, 0.510)
Marital status						
	Never married	ref	ref	ref	ref	ref
	Married/living with a partner	–0.849	0.577	0.145	0.428	0.428 (0.136, 1.349)
	Widowed/divorced/separated	–0.203	0.573	0.724	0.816	0.816 (0.261, 2.552)
Complicated with hypertension						
	No	ref	ref	ref	ref	ref
	Yes	–0.387	0.339	0.256	0.679	0.679 (0.346, 1.332)
Asthma						
	No	ref	ref	ref	ref	ref
	Yes	1.082	0.296	<0.001	2.952	2.952 (1.637, 5.321)
Anemia treatment in the past 3 months						
	No	ref	ref	ref	ref	ref
	Yes	0.671	0.463	0.151	1.955	1.955 (0.779, 4.906)
Overweight						
	No	ref	ref	ref	ref	ref
	Yes	0.552	0.295	0.064	1.737	1.737 (0.967, 3.121)
Arthritis						
	No	ref	ref	ref	ref	ref
	Yes	1.36	0.286	<0.0001	3.896	3.896 (2.205, 6.881)
Congestive heart failure						
	No	ref	ref	ref	ref	ref
	Yes	0.687	0.263	0.011	1.988	1.988 (1.177, 3.355)
Stroke						
	No	ref	ref	ref	ref	ref
	Yes	0.819	0.326	0.014	2.268	2.268 (1.187, 4.335)
Emphysema						
	No	ref	ref	ref	ref	ref
	Yes	0.239	0.307	0.437	1.27	1.270 (0.690, 2.337)
Chronic bronchitis						
	No	ref	ref	ref	ref	ref
	Yes	0.926	0.268	<0.001	2.524	2.524 (1.482, 4.297)
Liver diseases						
	No	ref	ref	ref	ref	ref
	Yes	0.294	0.503	0.561	1.341	1.341 (0.494, 3.644)
Thyroid problem						
	No	ref	ref	ref	ref	ref
	Yes	0.086	0.3	0.775	1.09	1.090 (0.600, 1.978)
Sleep disorder						
	No	ref	ref	ref	ref	ref
	Yes	1.834	0.254	<0.0001	6.258	6.258 (3.774, 10.376)
Complicated with diabetes						
	No	ref	ref	ref	ref	ref
	Yes	0.574	0.292	0.052	1.776	1.776 (0.995, 3.171)
Work restriction						
	No	ref	ref	ref	ref	ref
	Yes	1.999	0.32	<0.0001	7.379	7.379 (3.904, 13.948)
Walking disorder						
	No	ref	ref	ref	ref	ref
	Yes	1.288	0.282	<0.0001	3.626	3.626 (2.068, 6.359)
Drink wine/alcohol						
	No	ref	ref	ref	ref	ref
	Yes	0.224	0.319	0.485	1.251	1.251 (0.663, 2.361)

BMI, body mass index; OR, odds ratio; CI, confidence interval; Std. error, standard error of the mean; AA degree, Associate of Arts degree, a two-year undergraduate academic degree.

### 3.3 Results of Weighted Multivariate Logistic Regression Analysis of Major Depression in the Development Group

Sixteen variables, identified as potential risk factors through weighted univariate logistic regression, were incorporated as independent variables, with the incidence of major depression among patients with CHD as the dependent variable. A weighted multivariate regression model was developed using the stepwise regression method based on the minimum Akaike information criterion (AIC). This iterative process sought to determine the most parsimonious model that best fit the data. The final model, with a minimum AIC of 482.96, included nine variables. Among these, the independent risk factors for major depression in patients with CHD were waist circumference, smoking status, arthritis, sleep disorders, and work restrictions. Detailed results can be found in Table 3.

**Table 3.** **Results of weighted multivariate stepwise logistic regression analysis of major depression in the development group**.

Characters		Estimate	Std. error	*p*-value	OR	95% CI
Age		–0.019	0.013	0.154	0.982	0.982 (0.957, 1.007)
Waist circumference		0.026	0.01	0.009	1.027	1.027 (1.007, 1.047)
Poverty–income ratio						
	<1	ref	ref	ref	ref	ref
	1–3	–0.263	0.395	0.508	0.769	0.769 (0.350, 1.690)
	>3	–0.853	0.496	0.090	0.426	0.426 (0.159, 1.145)
Gender						
	Male	ref	ref	ref	ref	ref
	Female	0.418	0.288	0.151	1.519	1.519 (0.856, 2.698)
Smoke						
	Daily	ref	ref	ref	ref	ref
	Some days	–1.327	0.746	0.079	0.265	0.265 (0.060, 1.172)
	Never	–1.056	0.319	0.001	0.348	0.348 (0.184, 0.657)
Arthritis						
	No	ref	ref	ref	ref	ref
	Yes	1.094	0.356	0.003	2.987	2.987 (1.471, 6.066)
Congestive heart failure						
	No	ref	ref	ref	ref	ref
	Yes	0.617	0.312	0.051	1.854	1.854 (0.996, 3.448)
Sleep disorder						
	No	ref	ref	ref	ref	ref
	Yes	0.991	0.264	<0.001	2.695	2.695 (1.591, 4.564)
Work restriction						
	No	ref	ref	ref	ref	ref
	Yes	1.122	0.324	<0.001	3.071	3.071 (1.609, 5.861)

OR, odds ratio; CI, confidence interval; Std. error, standard error of the mean.

### 3.4 Establishment of a Nomogram Model for the Risk of Major Depression in Patients in the Development Group

**Fig. 2.**
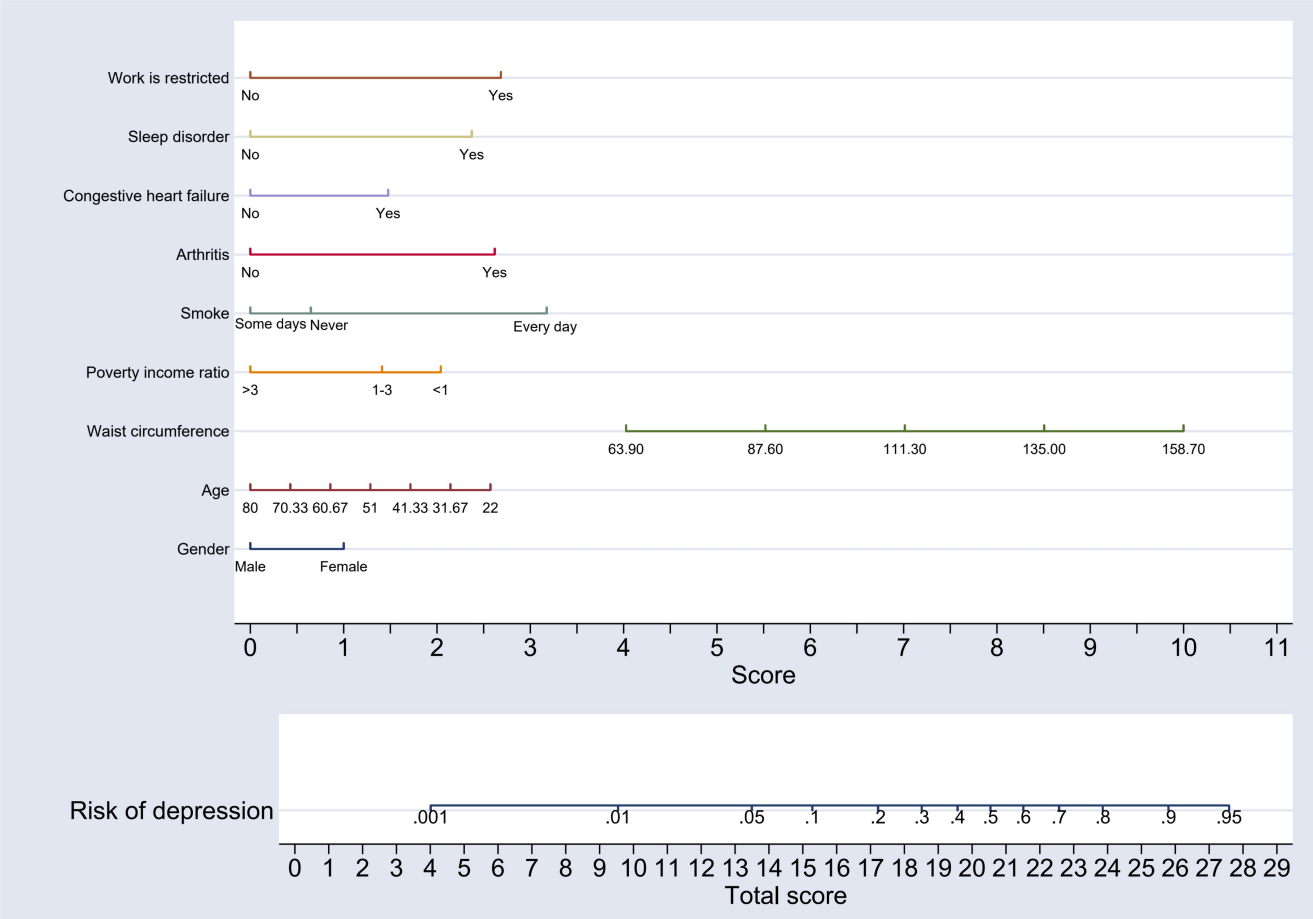
**Nomogram model for predicting the risk of major depression in patients in the development group**.

The predictive model developed using the minimum AIC stepwise regression analysis offers a key advantage in identifying variables with the greatest impact on prediction outcomes. This approach refines the model, enhancing both its accuracy and clinical applicability [26]. Consistent with practices employed in similar studies [27, 28] and drawing on the nine predictive indicators identified from the stepwise multivariate regression analysis based on the minimum AIC, a nomogram model was constructed to predict the risk of major depression within the development group. The model was developed using Stata 17.0, as illustrated in Fig. 2.

### 3.5 The Production of a Risk Score Table Based on the Nomogram Model

The nomogram interpretation is as follows: Each of the nine variables included in the nomogram was assigned a specific score, which can be determined by referencing the horizontal axis labeled “Score”. For each variable, the corresponding score can be identified by drawing a vertical line downward from the value of the variable. Once the scores for all nine variables are obtained, they are summed to calculate the “Total Score”. To estimate the risk, a vertical line is drawn upward from the horizontal axis labeled “Total Score” to the axis marked “Risk of Depression”. The value intersected on the “Risk of Depression” axis provides the predicted risk of major depression for patients with CHD. To facilitate accurate interpretation of the scores for each variable and to determine the major depression risk associated with the total score, a risk score table has been constructed as a supplementary tool to the nomogram. This table offers a clear and user-friendly reference for interpreting risk levels and is displayed in Table 4.

**Table 4.** **Risk score table based on the nomogram**.

Variables		Score
Age		
	80.00	0.0
	70.33	0.4
	60.67	0.9
	51.00	1.3
	41.33	1.7
	31.67	2.1
	22.00	2.6
Gender		
	Male	0.0
	Female	1.0
Waist circumference		
	63.90	4.0
	87.60	5.5
	111.30	7.0
	135.00	8.5
	158.70	10.0
Poverty–income ratio		
	<1	2.0
	1–3	1.4
	>3	0.0
Smoke		
	Daily	3.2
	Some days	0.0
	Never	0.6
Arthritis		
	No	0.0
	Yes	2.6
Congestive heart failure		
	No	0.0
	Yes	1.5
Sleep disorder		
	No	0.0
	Yes	2.4
Work restriction		
	No	0.0
	Yes	2.7
Risk of depression		Total score
0.001		4.0
0.01		9.5
0.05		13.5
0.1		15.3
0.2		17.2
0.3		18.5
0.4		19.6
0.5		20.5
0.6		21.5
0.7		22.6
0.8		23.8
0.9		25.8
0.95		27.5

### 3.5 Evaluation of Clinical Applicability of the Nomogram Model

**Fig. 3.**
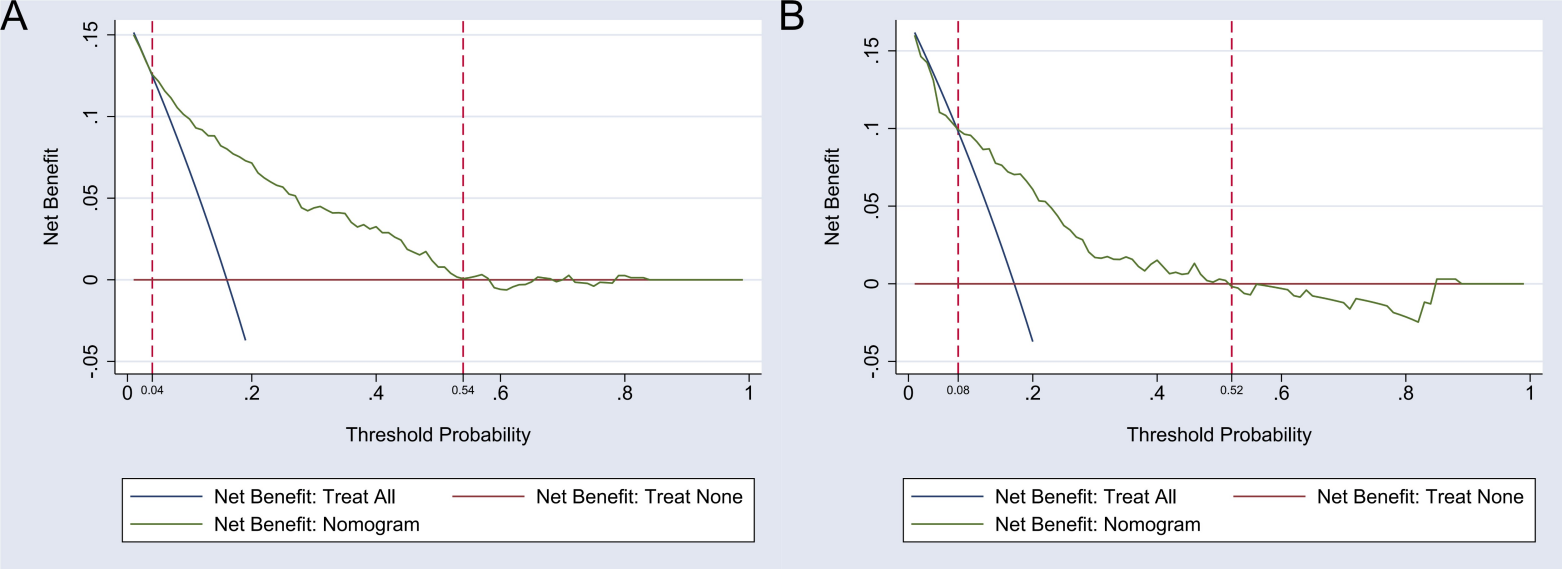
**Decision curve analysis (DCA) of the nomogram model**. Note: (A) The DCA of the nomogram model in the development group. (B) The DCA of the nomogram model in the validation group.

The DCA curve was employed to evaluate the clinical utility of the nomogram-based predictive model for major depression risk in the development group. The curve demonstrates that the nomogram achieves the highest clinical net benefit when the threshold probability for major depression in patients with CHD ranges from 0.04 to 0.54 in the development group and from 0.08 to 0.52 in the validation group. This performance significantly surpasses the “Treat All” and “Treat None” strategies, underscoring the strong clinical applicability of the nomogram. A detailed representation of these results is provided in Fig. 3.

### 3.6 Evaluation of Discrimination and Calibration of the Nomogram Model

**Fig. 4.**
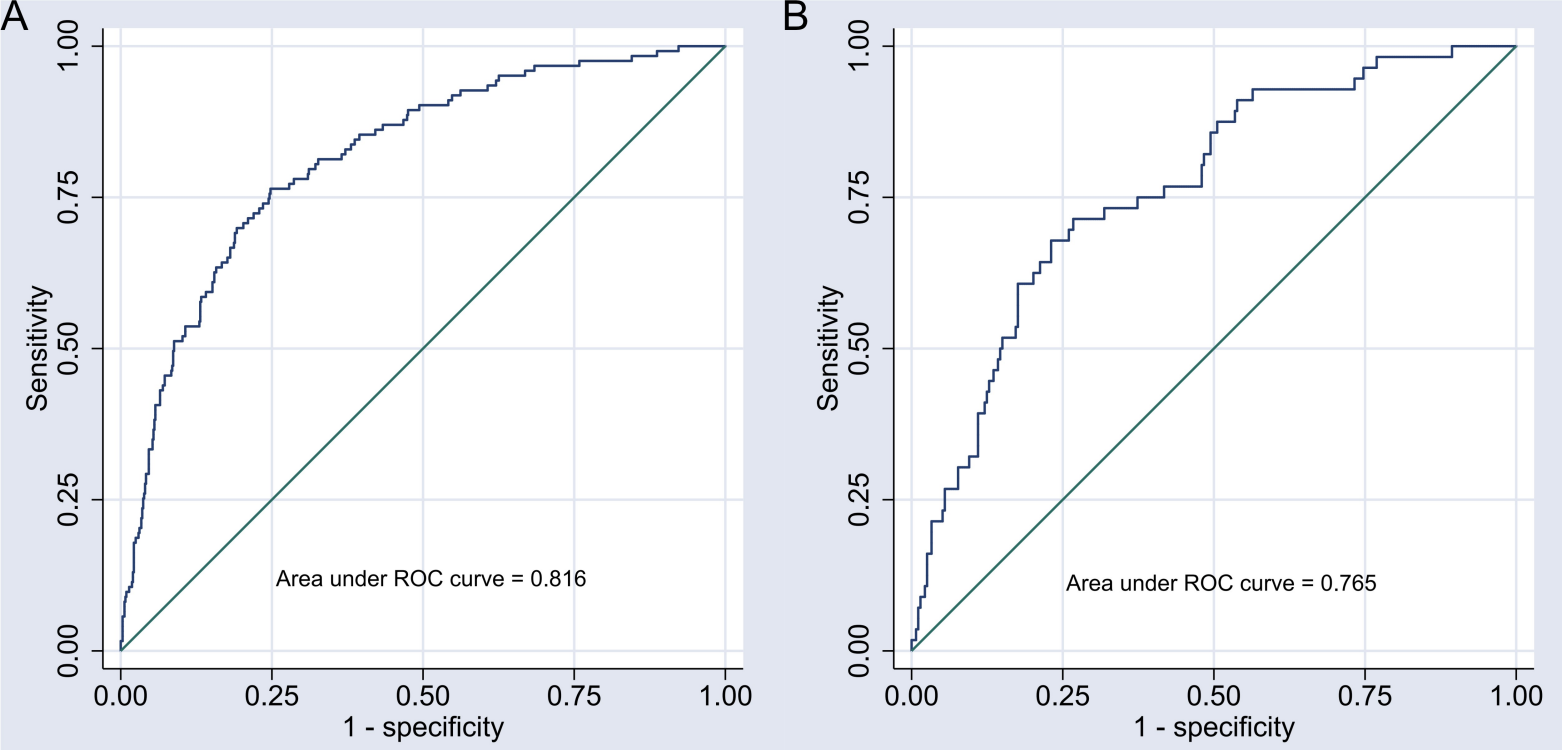
**Receiver operating characteristic (ROC) curve of the nomogram model**. Note: (A) ROC curve of the nomogram model in the development group. (B) ROC curve of the nomogram model in the validation group.

**Fig. 5.**
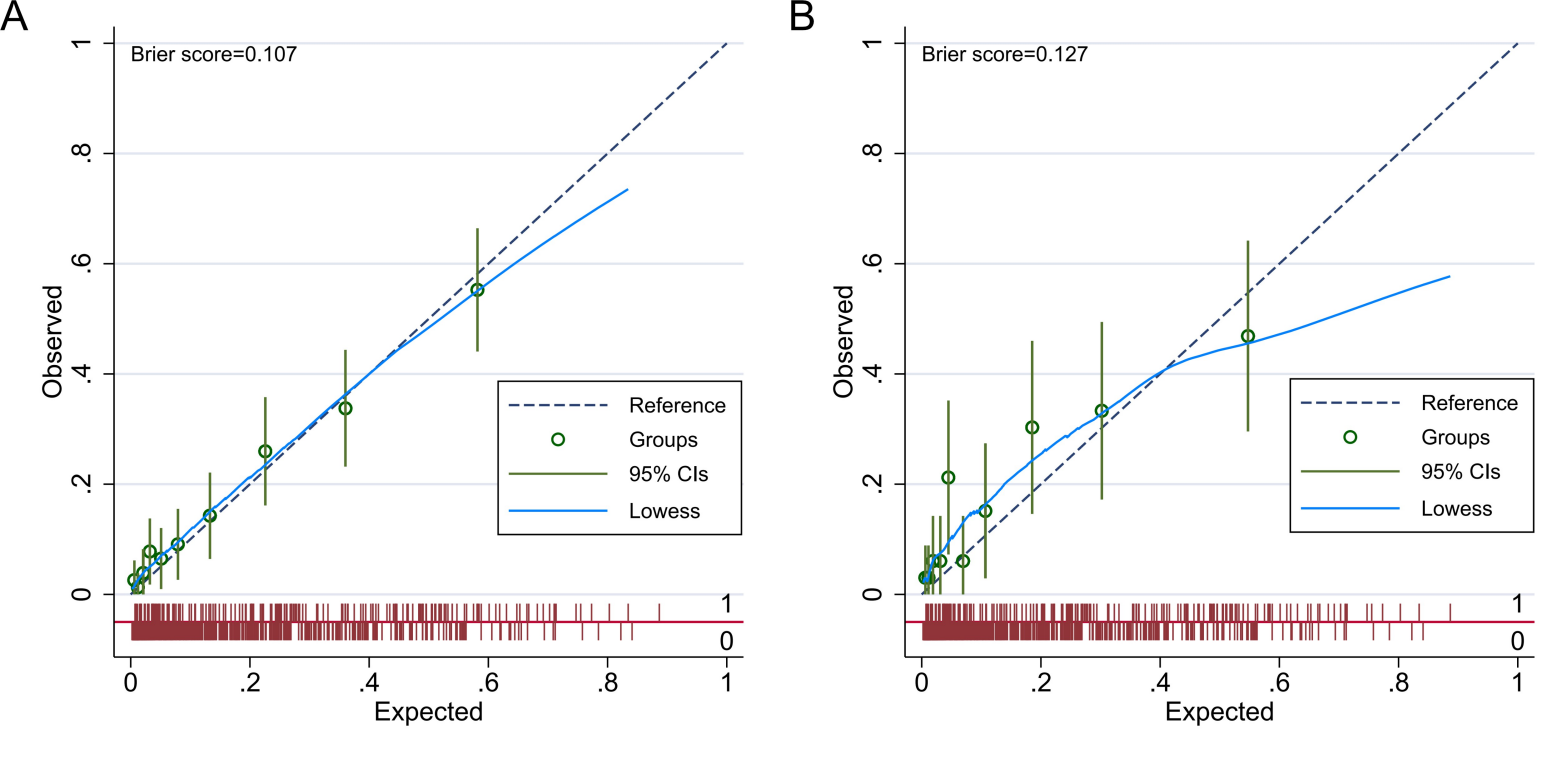
**Calibration curve of the nomogram model**. Note: (A) Calibration curve of the nomogram model in the development group. (B) Calibration curve of the nomogram model in the validation group. CI, confidence interval.

The areas under the ROC curve for the nomogram model were 0.816 (95% CI: 0.776–0.857) in the development group and 0.765 (95% CI: 0.699–0.832) in the validation group, demonstrating good discriminatory power. For further details, see Fig. 4. The Brier scores for the development and validation groups were 0.107 and 0.127, respectively, which are well below the 0.25 threshold, indicating a strong degree of calibration. This calibration analysis is illustrated in Fig. 5.

### 3.7 Validation of the Predictive Model for Different Levels of Depression Severity Using PHQ-9 Scores

To validate the predictive model for varying levels of depression severity, the performance of the model was evaluated using PHQ-9 scores of 5, 15, and 20 as thresholds, in addition to the established cutoff of 10 for major depression.

For a PHQ-9 score of 5, representing mild depression, the model showed an area under the ROC curve (AUC) of 0.810 in the development group and 0.797 in the validation group, demonstrating good accuracy in detecting individuals with mild depression.

At a PHQ-9 score of 15, indicating moderately severe depression, the model produced an AUC of 0.811 in the development group and 0.747 in the validation group, suggesting moderate effectiveness in distinguishing individuals with moderately severe depression.

For a PHQ-9 score of 20, representing severe depression, the model consistently achieved an AUC of 0.793 in both groups, indicating reliable identification of severe depression, a group at high risk for adverse outcomes.

These results highlight the predictive effectiveness of the model in categorizing different depression severity levels based on PHQ-9 scores. Furthermore, the consistent performance of the model across severity thresholds offers valuable insights for clinical decision-making and emphasizes the need for targeted interventions in patients with higher PHQ-9 scores.

## 4. Discussion

Owing to economic growth, improved living standards, and lifestyle shifts, the incidence of CHD has been steadily increasing [29]. Depression remains one of the most common psychological disorders among patients with CHD, with a prevalence of 27.6% in hospitalized patients with CHD in China [30]. This rate surpasses the 13.95% prevalence of major depression found in American patients with CHD, as revealed by this NHANES database analysis. Depression increases the risk of developing CHD in the general population by 1.5 to 2 times, while in patients with CHD, depression raises cardiovascular mortality risk by 2 to 4 times [31]. Moreover, depressive symptoms tend to persist over time [32]. Furthermore, individuals with CHD and comorbid depression experience more frequent chest pains, higher readmission rates, and a heightened risk of cardiovascular events, leading to a reduced quality of life and escalating healthcare costs [33]. Given the detrimental effects of depression in patients with CHD, identifying risk factors for depression in this population is essential. Such understanding would allow for targeted screening and preventive strategies, which can delay disease progression and improve patient outcomes.

This study, through weighted multivariate logistic regression analysis, identified waist circumference, arthritis, sleep disorders, and work restrictions as independent risk factors for major depression in patients with CHD, with odds ratios (ORs) exceeding one and *p*-values below 0.05. Notably, patients with CHD who had never smoked had a significantly lower risk of major depression compared to daily smokers (OR = 0.348, 95% CI: 0.184–0.657). These findings align with those by Gibson-Smith *et al*. [34], who emphasized waist circumference increases the risk of developing depression. As an indicator of abdominal obesity, waist circumference correlates with numerous health complications. The accumulation of visceral fat not only elevates cardiovascular disease risk but is also associated with chronic stress and depression, often resulting in reduced self-esteem and restricted social interactions, which negatively affect mental health. Previous studies have shown that obesity can exacerbate depressive symptoms [35, 36]. Moreover, arthritis is one of the common causes of physical pain and disability, which often makes patients fall into major depression [37]. Similarly, persistent sleep disturbances can disrupt circadian rhythms, causing mood disorders and mental health problems, including depression. Sleep disorders were found to be a modifiable risk factor in the development and maintenance of depression [38]. Work restrictions limit the ability of patients to engage in normal activities or maintain employment, leading to financial stress, social isolation, and reduced self-worth, all of which further elevate depression risk [22]. Additionally, smoking is a well-established risk factor for both cardiovascular disease and depression, with long-term smoking known to induce brain chemistry changes that increase the likelihood of depression. A prospective cohort study among Spanish college students also found that smoking was associated with an increased risk of depression [39].

A nomogram is a visual tool for clinical prediction that graphically represents the influence of each predictor on outcomes, providing a simplified, data-driven foundation for clinical decision-making [40]. Nomograms have previously proven useful in predicting depression risk, as evidenced by their application in patients with stroke [41] and obese state [42], where they have shown positive outcomes. However, no comprehensive nomogram predictive model currently exists in the literature for depression risk in patients with CHD. Thus, this study aimed to address this gap by constructing and validating a nomogram predictive model using NHANES data. Although the ORs for age, poverty–income ratio, gender, and congestive heart failure were not statistically significant (*p* > 0.05) in the weighted multivariate logistic regression, all nine indicators from the regression analysis were included in the nomogram. This decision was justified by the benefits of using the minimum AIC stepwise regression for model construction. The AUC in the model confirmed good discrimination, while DCA suggested strong clinical applicability, and the calibration curve demonstrated a high degree of calibration.

The nomogram in this study is user-friendly and, when combined with a risk score table, enables personalized risk assessments for patients with CHD, factoring in variables such as age and gender. Furthermore, the nomogram offers accurate predictions of major depression risk, providing clinical professionals with a tool to guide early intervention strategies, including psychological support, to reduce the likelihood of major depression.

### Limitations

Several limitations exist in this study that should be acknowledged: (1) While the NHANES database is nationally representative, the selection and participation of the sampled population may affect the generalizability of the model, particularly limiting its applicability to individuals outside the CHD population. (2) The nomogram model relies on lifestyle, behavioral information, and depression scores derived from self-reported questionnaires, which could introduce recall bias and affect the accuracy of data collection. (3) This study has been validated internally but lacks external validation, a critical limitation. While internal validation assesses model performance within the study sample, it does not provide insights into the model’s generalizability across diverse populations or clinical settings. Thus, the applicability of the model to different populations, such as various ethnicities, genders, or age groups, and clinical contexts remains uncertain without external validation. Incorporating external datasets for validation would greatly enhance the generalizability and utility of this model. (4) The model does not account for other potentially relevant predictors, such as laboratory markers, such as glucose metabolism, or lipid-related indexes, which could influence its predictive accuracy. (5) The nomogram model demonstrates correlations between the included indicators and depression risk in CHD but does not establish causal relationships. Further prospective research involving larger sample sizes and rigorous clinical designs is required to explore the extrapolation and generalizability capabilities of the model. (6) Additional sensitivity analyses, such as bootstrap methods, cross-validation, or penalized regression techniques, such as Lasso or ridge regression, could enhance the reliability and predictive performance of the model. (7) A more detailed subgroup analysis could clarify whether the risk factors for depression vary significantly by gender or ethnicity, offering more personalized insights and improving the model’s relevance to specific populations.

## 5. Conclusions

In summary, this study, utilizing data from the NHANES database (2005–2018), identified a 13.95% prevalence of major depression among patients with CHD. Multiple factors influence the onset of major depression in CHD. A nomogram model was developed based on the identified risk factors, demonstrating strong predictive accuracy and clinical applicability. This model can effectively identify patients with CHD at high risk for major depression, facilitating the implementation of targeted interventions. By addressing these high-risk individuals, this model may contribute to reducing the overall incidence of major depression in the CHD population.

## Availability of Data and Materials

The data used in this study is publicly available at https://www.cdc.gov/nchs/nhanes/index.htm.
